# Social validity of randomised controlled trials in health services research and intellectual disabilities: a qualitative exploration of stakeholder views

**DOI:** 10.1186/1745-6215-12-144

**Published:** 2011-06-09

**Authors:** Dan Robotham, Michael King, Anton Canagasabey, Sophie Inchley-Mort, Angela Hassiotis

**Affiliations:** 1 Mental Health Foundation, Sea Containers House, 20 Upper Ground, London SE1 9QB, UK; 2 Department of Mental Health Sciences, University College Medical School, Royal Free Hospital, Pond Street, London NW3 2QG, UK; 3 South Essex Partnership University NHS Foundation Trust, Learning Disability Psychiatry Department, 14 Tye Common Road, Billericay, Essex CM12 9ND, UK; 4 Camden Learning Disabilities Service, Bedford House, 125 Camden High Street, London, NW1 7JR, UK; 5 Department of Mental Health Sciences, University College Medical School, Charles Bell House, 67-73 Riding House Street, London W1W 7EY, UK

## Abstract

**Background:**

Randomised controlled trials (RCTs) are the gold standard of evidence-based practice in medicine but they have had limited influence in the field of intellectual disabilities. Previous literature suggests that participants and professionals have limited tolerance for this type of research methodology. However, it is not known how well service users, carers and other health professionals understand and accept the need for RCTs, and why it is important for individuals with intellectual disabilities to be included in this kind of research.

**Methods:**

We examined individual perceptions of RCTs in 51 participants (18 carers, 6 service users and 27 professionals) using semi-structured interviews. A framework approach was adopted in the analysis of data.

**Results:**

We found that participants had concerns about capacity and resource allocation but held positive views towards this type of research methodology. Understanding of the principles behind RCTs was poor amongst service users and a minority of carers, but mediated by previous exposure to research for professionals.

**Conclusions:**

The social validity of RCTs in intellectual disabilities may be compromised by lack of understanding of the design and the on-going concerns about obtaining informed consent especially in incapacitated adults. However, the overall finding that the need for this form of research was seen in a positive light suggests that there is a turning point in the perceptions of stakeholders working in intellectual disabilities services. We recommend that researchers include on-going education on RCT design during trials, tailoring it to all stakeholders with emphasis on strong service user and care involvement. This could be a pivotal element in improving acceptability of, and recruitment to RCTs.

## Background

It is estimated that there are 187,000 adults known to intellectual disability services in the UK [1]. Clinical research with this population has historically been problematic as it raises severe and at times intractable ethical concerns [2,3]. Inexperience with clinical research and randomised controlled trials (RCTs) in particular, may hinder the development of effective interventions for people with intellectual disabilities and mental disorders and/or challenging behaviour.

RCTs that include individuals with learning disabilities are rare, and only a small proportion of all papers published in specialist journals for intellectual disabilities concern RCTs [4-6]. The RCTs that have taken place have been limited by under-recruitment [7-11] or high drop-out rates [12]. Researchers have previously reported numerous barriers to conducting RCTs that include difficulties in communication, accessing participants through gatekeepers such as paid carers, lack of understanding about clinical trial processes amongst care agencies, and obtaining informed consent from service users [13-15]. The latter is particularly important in the UK in light of the Mental Capacity Act (2005) [16] which not only presumes capacity from the outset but also seeks to ensure that every effort is made for service users to receive appropriate support in order to make an informed decision.

There has been suggestion that stakeholders have limited tolerance for this kind of research and may be hostile towards RCTs therefore decreasing the likelihood of them participating in research trials [15,17]. In response to this notion we reviewed the literature available on participant experiences of RCTs [17] and found that stakeholders may be better able to understand the clinical equipoise than was previously thought and that stakeholder opinions may be changing. For example, parents of children with autism involved in drug RCTs showed that they had understood the various elements of a clinical trial such as the use of placebo, the need to test the medication efficacy, potential harm and benefits and the right to withdraw at any time [18]. Another questionnaire survey reported high levels of satisfaction with the practicalities relating to the use of drugs in autism and the conduct of the trial [19-21]. Importantly, the level of satisfaction reported was not related to participant's clinical outcome after the trial [21]. Furthermore, service users with mild intellectual disabilities may have greater understanding of RCT methods than might be expected [22]. These examples illuminate a potential discrepancy between carers and service users, and researchers who believe that RCTs in this population are inherently difficult.

Additionally, the Mental Capacity Act (2005) [16] presents a statutory framework which enables service users the opportunity to be involved in research and sets out the legal duties of researchers who wish to investigate any vulnerable population who may lack the ability to make a voluntary, informed decision. In cases where service users are deemed to lack capacity, 'best practice' states that proxy decision makers are essential, but that any decision made on behalf of someone without capacity must reflect the best interests of the individual concerned. The Act states that 'informed' consent should be gained for any individual to participate in research. Therefore service users and proxy decision makers should have a richer understanding of RCTs than has previously been assumed; else this would highlight an ethical issue and a problem relating to the RCT recruitment process.

It has been suggested that individuals are more likely to participate in a trial if they deem the research as being meaningful [21] but little work has been carried out to explore the "social validity" [23] of RCTs in this population. The concept of social validity originates in Applied Behaviour Analysis and focuses on whether the treatment goals of the intervention employed and the outcomes achieved are acceptable, relevant, and useful to the individual in treatment [19]. In order to enhance ethical quality and future research activity, whilst improving the evidence base for interventions for people with intellectual disabilities, it is important to explore stakeholders' perceptions of RCTs and the procedures that they may be asked to be involved in. Stakeholders may include service users, paid carers, family carers and professionals from health and social care background. Using qualitative, semi-structured interviews we aimed to investigate the opinions of stakeholders who were involved in a recent pragmatic RCT in intellectual disabilities concerning a psychological intervention for challenging behaviour. Pragmatic RCTs evaluate the comparative efficacy of two types of treatment in real life settings, i.e. not under experimental conditions [24]. Furthermore, pragmatic trials require wide inclusion criteria in order to gain results that can be generalised to the wider population. This is therefore particularly useful when including individuals with intellectual disabilities in RCTs as the population of people labelled as having intellectual disability includes a wide variety of individuals, with and without additional diagnoses as well as, a wide range of other individual differences.

## Methods

### Setting

The Randomised Evaluation of a Behaviour Intervention for Learning Disabilities (REBILD) aimed to investigate the clinical and cost effectiveness of a treatment model for adults displaying challenging behaviour. Applied Behavioural Analysis was delivered by a specialist team in addition to treatment as usual (TAU), and TAU consisted of assessment and management of service users by a variety of professionals such as occupational therapists, nurses or psychiatrists who populate community based intellectual disabilities teams in the UK. Sixty-three participants were recruited in total; thirty-two were allocated to the treatment and TAU arm and thirty-one to TAU. Details of the original clinical trial have been reported elsewhere [25]. We asked stakeholders including participants and professionals associated with the RCT to take part in the present project.

### Participants

We conducted semi-structured face to face qualitative interviews with 51 participants: six service users with mild intellectual disabilities who had adequate communication skills and were able to provide informed consent, 11 paid carers, seven mothers (family carers) of service users and 27 health and social care professionals. The majority of the overall sample was female (75%).

Two of the service users who were interviewed for this study had been randomised to TAU and four to the treatment plus TAU arm. Eight family or paid carers were part of a service user-carer dyad allocated to the control group and 10 had been allocated to the intervention group. Paid and family carers and service users were sampled after the final follow up for the RCT at six months. The interview timeframe was one day to three months post trial bar one carer who was interviewed after 10 months. Purposive sampling of health and social care professionals aimed to achieve maximum variation for the different professions within multi-disciplinary teams. We obtained a copy of the health and social services register for staff working in local intellectual disability services and recruited accordingly. The professionals included nurses and social workers of various grades, psychologists, psychiatrists, occupational therapists, speech and language therapists, and community support workers. Further details about the socio-demographic characteristics for all participants are given in table 1.

**Table 1:** Socio-demographic characteristics of participants

	Professional (n = 27)	Carer (n = 18; paid = 11, family = 7)	Service User (n = 6)	Total (n = 51)
	**N (%)**			

**Gender**				
Male	9 (33.3%)	1 (5.6%)	3 (50.0%)	13 (25.5%)
Female	18 (66.7%)	17 (94.4%)	3 (50.0%)	38 (74.5%)
**Age group**				
22-25	0 (0.0%)	1 (5.6%)	0 (0.0%)	1 (2.0%)
26-30	3 (11.1%)	1 (5.6%)	3 (50.0%)	7 (13.7%)
31-40	5 (18.5%)	1 (5.6%)	3 (50.0%)	9 (17.6%)
41-50	7 (25.9%)	2 (11.1%)	0 (0.0%)	9 (17.6%)
51-60	10 (37.0%)	11 (61.1%)	0 (0.0%)	21 (41.2%)
61+	2 (7.4%)	2 (11.1%)	0 (0.0%)	4 (7.8%)
**Experience (years)**				
	Mean = 17.6 yrs	Mean = 13.4 yrs		
1-5	4 (14.8%)	1 (5.6%)	-	-
6-10	5 (18.5%)	3 (16.7%)	-	-
11-20	6 (22.2%)	5 (27.8%)	-	-
21-30	7 (25.9%)	2 (11.1%)	-	-
31+	4 (14.8%)	0 (0.00%)	-	-
			-	-
**Employment sector**				
Health care	n=20	-	-	-
Nursing role	15	-	-	-
Psychiatrist	2	-	-	-
Psychologist	1	-	-	-
Speech therapist	1	-	-	-
Occupational therapist	1	-	-	-
Social Care	n = 7	-	-	-

### Instruments

Interview schedules for the carers and professionals were developed through reference to prior literature, and piloted. Topics included in the interview schedules related to research, RCTs, communication and informed consent. Accessible versions of the service user interview schedule and a vignette based on the REBILD trial were developed with assistance from speech and language therapists. Interview schedules consisted of open-ended main questions and probes. Probes served two purposes; firstly they enquired about areas of particular relevance. Secondly, they served to remind participants of the REBILD trial and elements therein should there be problems in recall. All interviews were audio taped except for two where the service users preferred that written notes be taken.

### Ethics

Ethical approval was given by Essex 1 (formerly West Essex) Research Ethics Committee prior to commencing the research project.

### Data Analysis

The audio taped interviews were transcribed verbatim and then checked for accuracy. Identifying information was removed to preserve anonymity and then transcripts were transferred to a qualitative research software programme [26].

A "framework" approach was used to analyse the interview data. It was coded through distinct coding rounds with six transcripts coded per round. A code summary was produced after each round. Similar codes were then merged together producing a second code summary. The summaries represented the first stage of framework development. The approach followed the 'Template Analysis' by King [27]. After each coding round, and as new data was analysed, networks of hierarchical relationships between codes were created and refined. Data saturation was estimated by counting the number of new codes and categories generated after each coding round; this was reached after the seventh round.

We ensured that the study complied with the four components of study "trustworthiness": credibility, confirmability, transferability and dependability [28]. Credibility was addressed by summarising interviews to interviewees and asking confirmatory questions. Confirmability was addressed by randomly selecting a sample of transcripts to be read by a second reader who coded them using the framework developed by the first reader. The two readers then discussed any differences. This was carried out twice, after the second and final coding rounds. The coding framework was revised until it could account for the complete data set. Anomalous data was identified during analysis. Transferability was addressed by purposive sampling, and recording contextual and demographic information. Dependability was addressed by keeping a diary of events and recording researcher perspectives. Analysis of pilot interviews identified that a less structured, more responsive approach was required and therefore methodology and analysis strategies were reactive to study context.

## Results

A number of common themes arose between the stakeholders groups. There was a perception amongst carers and professionals that insufficient financial resources were available to optimally support this population. However, despite this, views on research were almost unanimously positive especially when the research was designed to have a practical purpose. Professionals often expressed the view that service users' communication skills and ability to provide informed consent made the conduct of research ethically complicated. Below, we report on the most common themes that arose from each stakeholder group but a full list of all the themes we encountered in the narratives can be seen in table 2.

**Table 2:** Emerging themes and frequency per participant group

Theme	Service Users	Carers	Professionals	Total
Safeguarding	1	1	17	19
Motivation	3	14	3	20
Disadvantage/labelling	3	6	12	21
Fairness	5	16	13	34
Access	4	9	24	37
Funding/resources	2	13	23	38
Seeking help and support	4	16	19	39
Research outcomes	3	15	23	41
Research within services	1	15	27	43
Communication/understanding	5	13	25	43
Preferences	3	15	25	43
Perception of clinical research	4	16	24	44
Method	5	14	27	46
Informed consent	5	16	26	47
Benefits	5	16	27	48
The work environment	3	18	27	48
Terminology	5	17	26	49
Approach	5	18	27	50
Opinions about research	6	18	27	51

### 1. Service users

Irrespective of group allocation, a minority of the interviewed service users demonstrated some understanding of basic concepts and at least one reported that trials can be used to test the effectiveness of the service.

"[You're doing this research] just to see how well the service is run from a scale of one to ten" (Service user #6)

"Research is finding out stuff" (Service user #3)

Most service users focused on the fact that research required to "*ask for people's opinions*". Only one service user appeared unable to explain what research was in any way, describing it as "*something that gets you around*".

Service users were aware of the importance of communication skills in relation to their daily lives. One service user indicated that people with intellectual disability can develop complex skills, such as sign language, in order to compensate for communication deficits. They suggested the use of pictorial information to aid understanding.

"A couple of my friends are Down's and they can use sign language, they can lip-read as well. There's two of them, they're both very good at signs, very good. It's not easy to do or understand, I can't do it. Some people with learning disabilities can be very good at signs; it depends on how your brain works." (Service user #1)

"Using pictures might help" (Service user #1)

One service user was particularly positive about research and described it as a platform by which they could contribute. Another was particularly interested in finding out about the outcomes of the research.

"I think research is good because you get input off everyone else who...you have input in the fact that you can get your point across...to the researcher, then they can do the research on any information at the end of it, it's really quite a good input. You feel like you're giving something to the researcher." (Service user #5)

"I'd like to know where does this go, what becomes of this when it is completed. I just wondered really." (Service user #6)

Procedures such as chance allocation and comparison groups appeared to cause two service users worry about the 'fairness' of a procedure in which some participants were seen by a service before others.

"I don't think it should be chosen by a computer I think people should actually go through more...who needs the help the most and then put them at the top. [...] I think everyone should really get the help, I don't think you know, otherwise it isn't fair." (Service user #3)

"Six months is too long to wait for help. Within that time, those other ten that didn't get the help six months before were now probably...in and out of hospital, lost, don't know where they're going." (Service user #5)

"It really depends on if they see one party before the other, because it could be unfair to do the other people if they didn't get that treatment. That's just my opinion. I think everyone has got the right to equal opportunities and get services." (Service user #6)

One service user associated the allocation of participants to the treatment and TAU groups to the lack of resources in the local area, which was a perception shared across the participant groups.

"I think it's because there isn't enough nurses out there...there isn't enough nurses, there isn't enough...help groups, there isn't...to manage, there's a lot of patients out there that need help." (Service user #5)

### 2. Family carers

As with the service users, family carers were positive about research irrespective of the trial arm the service user was assigned to. There was greater emphasis on how the research would be followed up with practical action and dissemination.

"I think it's a good idea, it depends if it's just research or if they're actually gonna do something with it" (Family carer #3)

"It would be nicer to sort of, so that that you knew exactly the end result, what was actually going on with the research." (Family carer #5)

"If we didn't have research we wouldn't be where we were today would we? [...] if research wasn't done the scientists and things didn't do what they've gotta do we'd never...go out...you know get further on in medicine and whatever." (Family carer #6)

Family carers appeared to have a varied understanding of the trial procedures and were concerned that it might prevent access to services that they needed. Those who did not understand the trial procedure were unsure about why their relative had been allocated into the TAU group. However, one carer was able to describe the need for comparison groups in order to evaluate the service.

"Otherwise what have you got to compare it with? You've got no comparison. If everyone gets the same...service, if everyone got say both, if everyone just got the behaviour therapy service, then what have you got to compare it against? You've got nothing." (Family carer #2)

"It seems very random to pick them by computer as to say one...Group 1 or Group 2. I don't quite see the point of it [...] I don't understand. Why do they have two groups? I mean why are they not all assessed the same?" (Family carer #1)

"I didn't understand what it meant, I thought that once [the nurse] had put her information over to them that they would automatically get involved with X...and it didn't work out that way in the end." (Family carer #4)

The family carers were aware of the process of informed consent and questioned how the service users' communication problems might impact on that process. Mostly they felt it was natural to provide permission to participate on behalf of someone who lacked capacity. However, one family carer reported discomfort in taking responsibility for agreeing on behalf of a relative; she argued that her views were only one of many including other stakeholders (e.g. professionals) whose input was equally valid, but might contradict her own views.

"We are and were his consent on this, but we knew that it was for his good, and that why we at the...but whether it was the behaviour therapy, whether it was the community learning, that meant no difference to X...himself, it wouldn't have mattered whether it was the Pope." (Family carer #2)

"I wouldn't just go by the carer and parent because again it's a very, very sticky and a very awkward situation. I think you've got to have an input from everybody, psychiatrists, doctors, consultants but I do feel the perhaps 60-70% of the results should come through the carers." (Family carer #5)

### 3. Paid carers

Paid carers thought of research as important for the improvement of future service delivery.

"If you don't do research how are you going to learn? And make it better for the next lot that come along" (Residential care manager #1)

"Without research you're not finding out new information and you can't make your way forward can you?" (Paid carer #1)

"I have a positive attitude towards research on people with learning disabilities because I read a book where there were very many positive examples of how the research changed the work practice for the better of this client group." (Paid carer #2)

Paid carers had better understanding of the trial than family carers and service users. Several were aware of the use of placebo as control in drug trials, that usually there is a comparison group and, within this context, our trial procedures were seen as acceptable. More than family carers, some paid carers expressed doubt whether quantitative measurements used within RCTs including REBILD were subtle enough to measure the complex nature of each service user's difficulties.

"How else would you do it?...Because you've got to get...a different perspective haven't you? For the group that has been helped and the group that hasn't, it's a bit like taking a placebo innit?" (Residential care manager #2)

"As I say the questionnaires they're very good but they're questionnaires and questionnaires are never, never accurate because you can only say 'yes', 'no', 'maybe', 'sometimes'." (Paid carer #3)

However, two paid carers criticized the research process. For example, for one of the carers assessments carried out by the researcher were seen as potentially influencing the service user's allocation to the treatment group while the other likened the trial procedure to a lottery.

"My understanding was that you came here, took back as much details about the incident of the concerns we had with X, then you would then feed it back to the main core, the main centre then it was from that that they would decide whether the input or who would be selected." (Residential care manager #3)

"I really don't know, I've no idea...it's like picking your numbers out of the lottery...and, it's just not right." (Paid carer #3)

A foremost concern for the paid carers was the way in which consent was gained to enter a service user without capacity into a trial; paid carers liked a shared decision to be reached for those individuals. Subsequently, paid carers often believed that a decision should be made in the best interest of the service user.

"I think the whole team, if the person with the learning disability doesn't understand or won't agree, the whole team have to get together and find out, you know how we're gonna work around it." (Day services manager #1)

"I think a good consent procedure would be the key people working with the client himself or their selves, the people that are important to the client in their lives, and I think they are the best people to be able to make that decision but I don't mean doctors, consultants and people like that who only sort of see them for maybe 10 minutes, 15 minutes in a month." (Residential care manager #3)

### 4. Professionals

This group recognised that the area of intellectual disabilities was lacking in evidence based care that would be important for the development of treatments in this field. The professionals felt that evaluation of services was urgently needed, and in their interviews they tended to focus upon research outcomes rather than research process. As a group, they thought that it was important to disseminate research and to learn from it.

"It's a very important area for the obvious reasons that learning disabilities services have progressed so much in the last twenty to thirty years that...and again there has been very little research apart from in very key areas." (Nurse #1)

"Yeah I mean as long as it isn't...research for the sake of research, if there's a goal and it's gonna move the service on, fine, you know." (Social care professional #1)

"The main one [aim] is to do the research to see in what way we can improve the service and if possible get that published so that would disseminate the findings to other professionals." (Nurse #2)

Compared to the other stakeholders, professionals showed the greatest knowledge and understanding of the rationale for undertaking a RCT (in order to evaluate a treatment or service). Some professionals, particularly psychiatrists, were enthused about the clinical trial and considered randomisation as an essential component in increasing the reliability of the results.

"Well there's nothing to think about, randomised controlled trials are important, important to find out and...how effective the treatment that is helping people." (Psychiatrist #1)

"I quite understand that without doing it, we wouldn't know the result. It's one of those things that I believe has to be done in order to get some answers" (Nurse #1)

Other professionals agreed with the value and need for the trial but felt frustrated because their preference would have been for the service users to access such a highly regarded service as quickly as possible. The biggest problem identified was the perceived interference with referrals to the specialist service as a result of the trial which led to participants expressing dissatisfaction with the project. At times, the trial was viewed negatively because of this. However, there was an accepted six month waiting list at the time that the trial commenced.

"By randomising the cases, it's stopping us from using the service that we really badly need, because we're not just referring for the sake of referring, we've tried everything we can." (Nurse #4)

"You need a control group for comparing it against and the only fair way of doing that is to randomise it...But yeah it has just been frustrating with lots of our clients being under the control group." (Professional therapist #3)

Professionals also expressed doubt about whether service users would understand the complexities of the trial.

"I don't think a lot of our clients could ever understand the implications of something like this." (Nurse #5)

"It has always been a difficult field because you do not have a subjective view of the service user [...] I find it quite difficult to undertake research especially where the service users are...involved." (Nurse #6)

As a group they also thought that multidisciplinary consent from various sources was necessary in order for service users to take part in the trial.

"It's obviously a complex matter, however I feel that a multi-disciplinary type of community or team to offer consent in those cases, appropriate in people from professions, their family and as well as advocacy service." (Psychiatrist #1)

"We need to have a multi-disciplinary team meeting to see whether it is for the benefit or for the goodness of the client. In other words you can get the consent from the multi-disciplinary team on behalf of the clients." (Nurse #3)

## Discussion

### Main findings

The majority of stakeholders viewed the methods underpinning RCTs and the need to conduct such research in a positive light. Therefore, the social validity of such research appears to have improved considerably in recent years compared with previous reports.

RCT procedures were seen in a mixed light. Firstly, it is important to note that trial procedures such as random allocation were poorly understood within the service user and family carer groups. Clinical training and experience unsurprisingly appeared to affect understanding in a positive way. Acceptance of the trial in professional and paid carer groups therefore appeared to be influenced by their acceptance of research outcomes, and of the perceived need to engage in research.

When prompted, some of the service users interviewed wondered about the fairness of random allocation to the two groups. Similarly, family carers were concerned about what the individual service user stood to gain from the research. For professionals, the emphasis was often on improving services or at least not compromising current care. They expressed positive views about the trial as long as it could help to make progress in the long term; however, negative opinions appeared to be associated with an anxiety about trials being a door to diverting resources away from highly prized services. A practical consideration for stakeholders, as expressed by some professionals, was of barriers to accessing specialist treatment and their frequently expressed preference for the Specialist Behaviour Service over TAU; they had preferences for a new treatment over an old one. This contradicts the idea of clinical equipoise, where a clinical community is uncertain about whether one treatment is more effective than another, and there is insubstantial evidence to suggest that one treatment is superior. In this context however, participants might benefit from accessing treatments that would otherwise be unavailable to them because of the scarcity of resources and the length of waiting lists, at least half of the time.

Carers and professionals expressed significant worries about obtaining valid consent and the ethics of deciding on someone else's behalf. Most professionals and one paid carer suggested that multidisciplinary teams should meet to discuss participation in RCTs or research in general and whether this was in the service user's best interests.

### Strengths and weaknesses

This is the first qualitative study to explore the views of a diverse range of individuals who have participated in a pragmatic RCT that involved people with intellectual disabilities. The stakeholders came from a wide range of settings and socio-demographic backgrounds across the intellectual disability community. The qualitative analysis strategy led to a more detailed description of context and a greater degree of flexibility in interpreting the data. The setting was similar to that of community intellectual disabilities services across the UK. Although it can be argued that the results reflect a local situation, the concerns and opinions expressed are by no means restricted to the service users with intellectual disabilities but are widespread across other populations and interventions. We would have liked to have recruited more service users but the condition that individuals had to have taken part in the REBILD study and have sufficient communication abilities to be interviewed, limited the number of service users that were eligible. As it is, 30% of all those eligible agreed to be interviewed. It would have been of particular interest to include those who had opted out of the RCT trial as it is possible that individuals who do not wish to enrol in RCTs might differ in their attitudes towards RCTs. However, there were only six individuals who declined to take part when first approached, therefore, we do not believe that our findings would be drastically altered. Furthermore, this particular RCT recruited people with intellectual disabilities and challenging behaviour, which meant that the service users may have been more difficult to work with than average, and the carers and professionals more desperate for help.

### Results in context

Many studies have focused on the tendency for patient and carer participants to misunderstand the methodological rationale behind an RCT. This has resulted in an extensive discourse about the concept of therapeutic misconceptions [29]. It suggests that RCT participants continue to believe that the treatment will be provided in accordance with their needs. In doing so, they fail to understand the implications of random allocation. It is suggested that increased understanding of trial concepts may lead to greater satisfaction with RCTs [30], which in turn is likely to increase participation in and engagement with trials.

Prior surveys of carers of people with intellectual disabilities sought to explore participants' satisfaction with RCTs [19-21] in order to demonstrate the acceptance or social validity of these types of trials. Similar to our study, researchers stated that carers were satisfied with and supportive of RCTs. However, these studies related solely to pharmacological clinical trials and not to psychological interventions. They also relied on quantitative multiple-choice format rather than in-depth interviews that explored the opinions of stakeholders towards the trial. Given the limited number of RCTs in the field of intellectual disabilities, our study not only adds to the previous body of work, but updates the often held belief that stakeholders are negative towards research in general and RCTs in particular in intellectual disabilities.

Recruitment is a recognised problem in many RCTs. Recent data suggest that out of 114 UK trials funded by the Medical Research Council and the Health Technology Assessment programme only 31% recruited successfully, 45% recruited less than 80% intended, and more than half required an extension [31].

A systematic review [32] of barriers to recruiting participants into RCTs in other fields reported similar concerns to those in our study. Examples are individual preferences to be allocated to the intervention over the control group, uncertainty about the outcome of the intervention, and concern about the process of obtaining informed consent. Elsewhere, it has been noted that members of the public would be more likely to enter RCTs for reasons of self-interest rather than altruism [33]. Individuals in this study showed preference for the intervention group suggesting that they were hoping to experience direct therapeutic benefit from the novel intervention and therefore, the present findings echo this previously noted observation.

### Meaning of the study

It appears that the increasing acceptability of RCTs is not merely dependent on the type of interventions, e.g. drugs vs. psychological therapies, but may herald a change in how practice and outcomes are currently regarded within the clinical and research community in intellectual disabilities. Despite full recruitment to the REBILD trial, low numbers of RCTs that include people with intellectual disabilities suggest that recruitment rates may be more atypical than the general population (personal communication to AH by Anna Cooper, 2010). In addition, the majority of service users with intellectual disabilities fail to fully understand the rationale behind an RCT. Detailed understanding of research methods by carers and professionals should not be assumed either and therefore it is imperative that researchers seek innovative and accessible ways in which to familiarise stakeholders with the RCT design concepts.

Our findings suggest several topics that researchers should address before they start a trial. For example, when discussing a study with service users or family carers it is important to consider how the study will affect participants personally, during and after the research, and how the activity will translate into practical outcomes. Additionally, when discussing a study with professionals, researchers should emphasise how the work will fit alongside existing patterns of service delivery and how it may advance local strategies for future service development. Furthermore, including service users in active roles as co-researchers or consultants during the design process has been found to improve research relevance, recruitment and dissemination [34,35]. This has been carried out in the field of intellectual disabilities research [e.g. 36-37]. Therefore, information sheets can be developed and presented in a way that makes the most sense to the service user [38] and studies show that they aid in obtaining informed consent [34]. Different types of communication aids for people with intellectual disabilities, such as Talking Mats, may assist in the service user expressing him/herself more freely, thus, overcoming communication challenges.

A report for the Service Delivery and Organisation programme stated that people with intellectual disabilities would like to take a bigger role in research [39] but little has been done in this respect regarding RCTs. Inclusive research can increase ethical quality and make research more understandable and relevant to the people that it ultimately affects and therefore this is one possible option for improving research in this area in the future.

## Conclusion

Our findings are in contrast to previous research [15] which argued that stakeholders in intellectual disabilities services were hostile towards or put up barriers to the conduct of RCTs in this population group. This is a new perspective that has implications for researchers in overcoming difficulties which include making research related information more accessible, understandable, and meaningful. We encourage researchers to consider all options to engage with the stakeholders and to evaluate these options given the widespread issues of poor recruitment and understanding of clinical trial methodology. The benefits of participation in research, although not immediately accrued, are significant given the widely acknowledged health and social inequalities that people with intellectual disabilities experience. Therefore it is timely to abandon long held views within the scientific community about stakeholder opposition to RCTs and ensure that the care people with intellectual disabilities receive is based on the best evidence available.

## List of abbreviations

RCT: Randomised Controlled Trial; REBILD: The Randomised Evaluation of a Behaviour Intervention for Learning Disabilities; TAU: Treatment as usual.

## Competing interests

The authors declare that they have no competing interests.

## Authors' contributions

AH, MK and AC conceived the study and DR wrote the protocol and carried out the data collection and analysis. SIM carried out additional literature reviews. All authors contributed to and approved submission of the final manuscript.

## References

[B1] Emerson E Hatton C Estimating Future Need for Adult Social Care Services for People with Learning Disabilities In England 2008 Lancaster: Centre for Disability Research, Lancaster University

[B2] Advisory Committee on Human Radiation Experiments The Human Radiation Experiments 1996 New York: Oxford University Press

[B3] Rothman DJ Rothman SM A Decade of Struggle for Social Justice The Willowbrook Wars 1984 New York: Harper & Row

[B4] Ashman L Duggan L Interventions for learning disabled sex offenders Cochrane Database of Systematic Reviews 2008 1 CD003682 10.1002/14651858.CD003682.pub2 PMC6991930 18254027

[B5] Beavis J Kerr M Marson AG Non-pharmacological interventions for epilepsy in people with intellectual disabilities Cochrane Database of Systematic Reviews 2007 4 CD005399 10.1002/14651858.CD005502.pub2 17943860

[B6] Hassiotis A Research in mental health learning disabilities: present challenges and future drivers Psychiatry 2009 11 457 460

[B7] Dowling S Hubert J White S Hollins S Bereaved adults with intellectual disabilities: a combined randomised controlled trial and qualitative study of two community-based interventions Journal of Intellectual Disability Research 2006 50 277 287 10.1111/j.1365-2788.2005.00759.x 16507032

[B8] Llewellyn G McConnell D Honey A Mayes R Russo D Promoting health and home safety for children of parents with intellectual disability: a randomised controlled trial Research in Developmental Disabilities 2003 24 405 431 10.1016/j.ridd.2003.06.001 14622893

[B9] Tyrer P Oliver-Africano PC Ahmed Z Bouras N Cooray S Deb S Murphy D Hare M Meade M Reece B Kramo K Bhaumik S Harley D Regan A Thomas D Rao B North B Eliahoo J Karatela S Soni A Crawford M Risperidone, haloperidol, and placebo in the treatment of aggressive challenging behaviour in patients with intellectual disability: a randomised controlled trial Lancet 2008 37 57 63 10.1016/S0140-6736(08)60072-0 18177776

[B10] Oliver PC Piachaud J Tyrer P Regan A Dack M Alexander R Bakala A Cooray S Done DJ Rao B Randomised controlled trial of assertive community treatment in intellectual disability: the TACTILD study Journal of Intellectual Disabilities Research 2005 49 507 515 10.1111/j.1365-2788.2005.00706.x 15966958

[B11] Willner P Jones J Tams R Green G A randomised controlled trial of the efficacy of a cognitive-behavioural anger management group for clients with learning disabilities Journal of Applied Research in Intellectual Disabilities 2002 15 224 235 10.1046/j.1468-3148.2002.00121.x

[B12] Kerr MP Baker GA Brodie MJ A randomised, double-blind, placebo-controlled trial of topiramate in adults with epilepsy and intellectual disability: Impact on seizures, severity, and quality of life Epilepsy & Behaviour 2005 7 472 480 10.1016/j.yebeh.2005.07.006 16140593

[B13] Lennox N Taylor M Rey-Conde T Bain C Purdie DM Boyle F Beating the barriers: recruitment of people with intellectual disability to participate in research Journal of Intellectual Disability Research 2005 49 296 305 10.1111/j.1365-2788.2005.00618.x 15816817

[B14] Oliver PC Piachaud J Done J Regan A Cooray S Tyrer P Difficulties in conducting a randomised controlled trial of health service interventions in intellectual disability: implications for evidence-based practice Journal of Intellectual Disability Research 2002 46 340 345 10.1046/j.1365-2788.2002.00408.x 12000585

[B15] Oliver-Africano P Dickens S Ahmed Z Bouras N Cooray S Deb S Knapp M Hare M Meade M Reece B Bhaumik S Harley D Piachaud J Regan A Ade Thomas D Karatela S Rao B Dzendrowskyj T Lenôtre L Watson J Tyrer P Overcoming the barriers experienced in conducting a medication trial in adults with aggressive challenging behaviour and intellectual disabilities Journal of Intellectual Disabilities Research 2009 54 17 24 10.1111/j.1365-2788.2009.01195.x 19627427

[B16] Mental Capacity Act 2005 http://www.opsi.gov.uk/acts/acts2005/ukpga_20050009_en_1

[B17] Robotham D Hassiotis A Randomised controlled trials in learning disabilities: a review of participant experiences Advances in Mental Health and Learning Disabilities 2009 3 42 46

[B18] Vitiello B Aman MG Scahill L McCracken JT McDougle CJ Tierney E Davies M Arnold LE Research knowledge among parents of children participating in a randomised clinical trial Journal of the American Academy of Child and Adolescent Psychiatry 2005 44 145 149 10.1097/00004583-200502000-00006 15689727

[B19] Aman MG Wolford PL Consumer satisfaction with involvement in drug research: a social validity study Journal of the American Academy of Child and Adolescent Psychiatry 1995 34 940 945 10.1097/00004583-199507000-00018 7649965

[B20] McAdam DB Zarcone JR Hellings J Napolitano DA Schroeder SR Effects of Risperidone on aberrant behaviour in persons with developmental disabilities: II. Social validity measures American Journal of Mental Retardation 2002 107 261 269 10.1352/0895-8017(2002)107<0261:EOROAB>2.0.CO;2 12069645

[B21] Tierney E Aman M Stout D Pappas K Arnold LE Vitiello B Scahill L McDougle C McCracken J Wheeler C Martin A Posey D Shah B Parent satisfaction in a multi-site acute trial of Risperidone in children with autism: a social validity study Psychopharmacology 2007 191 149 157 10.1007/s00213-006-0604-z 17123125

[B22] Fisher CB Cea CD Davidson PW Fried AL Capacity of persons with mental retardation to consent to participate in Randomised Clinical Trials American Journal of Psychiatry 2006 163 1813 1820 10.1176/appi.ajp.163.10.1813 17012694

[B23] Foster SL Mash EJ Assessing Social Validity in Clinical Treatment Research Issues and Procedures Journal of Consulting and Clinical Psychology 1999 67 308 319 10369051 10.1037//0022-006x.67.3.308

[B24] MacPherson H Pragmatic clinical trials Complementary Therapies in Medicine 2004 12 136 140 10.1016/j.ctim.2004.07.043 15561524

[B25] Hassiotis A Robotham D Canagasabey A Murad S Romeo R Langridge D Blizard R Murad S King M A randomised, single-blind, controlled trial of a specialist behaviour therapy team for challenging behaviour in adults with intellectual disabilities American Journal of Psychiatry 2009 166 1278 1285 10.1176/appi.ajp.2009.08111747 19687128

[B26] N6 Qualitative data analysis software 2002 Version 6: QSR International Pty Ltd

[B27] King N Symons G, Cassell, C London Template Analysis Qualitative Methods and Analysis in Organizational Research 1995 SAGE Publications 118 134

[B28] Lincoln YS Guba E Naturalistic Enquiry 1985 London: SAGE publications

[B29] Appelbaum PS Roth LH Lidz CW Benson P Winslade W False hopes and best data: consent to research and the therapeutic misconception Hastings Centre Report 1987 17 20 24 3294743

[B30] Heaven B Murtagh M Rapley T May C Graham R Kaner E Thomson R Patients or research subjects? A qualitative study of participation in a randomised controlled trial of a complex intervention Patient Education and Counselling 2005 62 260 270 10.1016/j.pec.2005.07.013 16181766

[B31] McDonald A Knight R Campbell M Entwistle VA Grant AM Crook JA Elbourne DR Francis D Garcia J Roberts I Snowdon C What influences recruitment to randomised controlled trials? A review of trials funded by two UK funding agencies Trials 2006 7 9 10.1186/1745-6215-7-9 16603070 PMC1475627

[B32] Ross S Grant A Counsell C Gillespie W Russell I Prescott R Barriers to participation in randomised controlled trials: A systematic review Journal of Clinical Epidemiology 1991 52 1143 1156 10.1016/s0895-4356(99)00141-9 10580777

[B33] Edwards SJL Lilford RJ Hewson J The ethics of randomised controlled trials from the perspectives of patients, the public, and healthcare professionals British Medical Journal 1998 317 1209 1212 9794861 10.1136/bmj.317.7167.1209 PMC1114158

[B34] Staley K Minogue V User involvement leads to more ethically sound research Clinical Ethic 2006 1 95 100 10.1258/147775006777254489

[B35] Hanley B Bradburn J Barnes M Evans C Goodare H Kelson M Kent A Oliver S Thomas S Wallcraft J Involving the Public in NHS, Public Health and Social Care Research: Briefing Notes for Researchers 2004 Eastleigh: INVOLVE

[B36] Walmsley J Johnson K Inclusive Research with People with Learning Disabilities: Past, Present and Futures 2003 London: Jessica Kingsley Publishers

[B37] Abell S Ashmore J Beart S Brownley P Butcher A Clarke Z Combes H Francis E Hayes S Hemmingham I Hicks K Ibraham A Kenyon E Lee D McClimens A Collins M Newton J Wilson D Including everyone in research: The Burton Street Research Group British Journal of Learning Disabilities 2007 35 121 124 10.1111/j.1468-3156.2006.00425.x

[B38] Robinson EJ Kerr CEP Stevens AJ Lilford RJ Braunholtz DA Edwards SJ Beck SR Rowley MG Lay public's understanding of equipoise and randomisation in randomised controlled trials Health Technology Assessment 2005 9 10.3310/hta9080 15763039

[B39] Williams V Marriott A Townsley R Shaping our Future: a scoping and consultation exercise to determine research priorities in learning disability for the next ten years 2008 London: NIHR/SDO

